# Financial Development and Health Outcomes: Do Financial Globalization Matter in Selected Asian Economies?

**DOI:** 10.3389/fpubh.2022.843935

**Published:** 2022-04-01

**Authors:** Guoxin Shi, Dawei Wang, Mehmet Altuntaş

**Affiliations:** ^1^School of Finance, Changchun University of Finance and Economics, Changchun, China; ^2^Northeast Asia Research Center of Jilin University, Changchun, China; ^3^Department of Economics, Faculty of Economics, Administrative and Social Sciences, Nisantasi University, Istanbul, Turkey

**Keywords:** financial development, financial globalization, health, China, India, Japan

## Abstract

The importance of health is well documented in the development economics literature because of its increasing effects on economic growth in the long-run. Financial development and financial globalization are essential resources for health. This study examines the role of financial development and financial globalization in the rapid rise of life expectancy in China, India, and Japan by using the annual data covering the period of 1991–2019. The ARDL bounds testing approach confirm the long-run relationship between financial development, financial globalization, and life expectancy in the presence of GDP, health expenditure, and the internet. The long-run findings indicate that financial development positively affects life expectancy by 0.599% in China. The novel findings also indicate that financial globalization positively affects life expectancy by 1.247% in Japan and 1.121% in India. Our findings offer new empirical insights to policymakers crucial to improving life expectancy in China, India, and Japan.

## Introduction

Life expectancy is used to represent a nation's health and used as a vital indicator to verify the development of a country by various international agencies. Therefore, the life expectancy rate can have long-lasting effects on the level of national income, fertility rate, transfer payments, pension schemes, and public finance (1, 2). Further, the progress and growth of some healthcare industries like insurance, pharmaceuticals, and aged care services rely heavily on life expectancy. Consistent with this view, in recent times, the importance of life expectancy has increased manifold in the eyes of empirics and researchers. They have started analyzing the economic, social, and environmental factors that can affect the level of life expectancy of a nation. Income, health spending, food obtainability, and pharmaceutical outlay are the crucial economic factors that can determine the national health status of a country. Among the social factors smoking, drinking, literacy rate, political conditions, and marital status are considered to be the vital determinants of the level of life expectancy. As far as environmental determinants of health status are concerned, urbanization, affluence, safety, industrialization, and regulation are noteworthy to be mentioned (1). Although many studies are available that have tried to detect the various determinants of longevity, very few studies have tried to analyze the relationship between financial development and health status.

There are various channels through which financial development can affect the health status of a nation (3). The first and foremost channel is the income effect. Financial development gives rise to industrialization and generates new economic activities that cause the level of employment and per capita income to rise. The rise in per capita income allows people to afford health and nutritional food, better housing, state-of-the-art hospitals, and health care facilities that positively impact the nation's health outcomes. Secondly, education is also an important channel that can positively affect life expectancy because financial development increases the literacy rate in society and, ultimately, life expectancy. Another important channel is the gender equality effect which says that along with the development of the financial sector in the economy, the women in the society become more financially independent. As a result, the level of women's empowerment in society also rises, and an empowered woman ranked the health status of the whole family as a top priority. Further, a financially independent woman spends more on her children's health than a man. Therefore, we can confirm that if women have easy access to financial services, it will improve the overall health status of a family and, eventually, the nation. Financial development improves population health by increasing health expenditure (4–7). Last but not least, financial development can improve health outcomes through the infrastructural effect. As already mentioned, financial development contributes to a nation's economic growth, which allows the public and private sector to invest more in building better hospitals and health care infrastructure, causing the life expectancy to rise and infant mortality to fall (8, 9).

On the other side, financial development may reduce life expectancy if a poor family requires excessive collateral to acquire financial services. To cope with high collateral, the poor family is bound to sell its valuable assets that cause their income to fall and, as a result, their health condition deteriorates. Besides, if the financial services are only available for the privileged class of the society, in that case, it leads to a financial crisis resulting in slow economic growth over a long period of time (10). In the time of financial crisis, the income of the government and household sharply declined, ultimately reducing their spending on health care programs, health insurances, hospitals, laboratories, and other health-related infrastructure, resulting in deteriorating health indicators in the country.

Over the past few decades, globalization has become the central focus of social scientists and international leaders. As a result, the impact of globalization on various economic and social indicators has been extensively studied. However, there are not many studies available that have analyzed the relationship between globalization and a country's health system (11, 12). Globalization simply refers to the situation where countries are closely connected and share their economic ideas, political platforms, and social traits (13, 14). According to Dreher (13), globalization aids the economies to grow at an incredible pace, suggesting that it is beneficial for the long-term economic development of the economy. Sirgy et al. (15) examined the link between globalization and life expectancy in developing economies due to the vulnerability of these economies to any health-related shock. Globalization reduces health inequalities in developing economies through various macroeconomic transmission channels (16). Though few studies have estimated the relationship between globalization and human health (17–20), the majority of them highlighted various channels through which globalization can affect human health (21). These channels include income, education, technology, and intake effects. The first two effects are already explained above and do not require further elaboration. The technological developments allow the use of modern and sophisticated technology in the healthcare industry to improve a nation's health standards (22). Lastly, the intake effects suggest that, because of globalization, people adopted a western lifestyle and started to consume fast food with high fat and sugar content, causing life expectancy to fall (23).

The above-mentioned literature proposes that the impact of financial development and globalization on health is not yet conclusive. Previous studies mainly focus on overall globalization ignoring financial globalization. To our information, no past study has explored financial globalization with health. The prior studies have only found the long-run effect of financial development and globalization on health ignoring short-run effects. Consistent with the views mentioned above, we aim to investigate the impact of financial development and financial globalization on the health outcomes of three Asian economies, namely China, India, and Japan. These three economies are among the largest economies of Asia and hold better healthcare infrastructure and systems among Asian economies. None of the past studies have targeted these economies while analyzing this relationship. Hence, this study is an effort to plug this gap into the literature. For empirical analysis, we have relied on the ARDL model, which is the best sample size model if small, and it provides short and long-run estimates simultaneously.

## Model and Methods

Following the health economies literature, the two most important determinants of health are financial development and financial globalization. This research work proceeds under a theory of neoliberalism, first familiarized by Friedrich Hayek in the 1920s. In doing so, we follow (3, 24) and adopt the following long-term specification:


(1) $$\begin{array}{l} Health_{t}=\;\varphi_{0}+\;\varphi_{1}FD_{t}+\varphi_{2}FG_{t}+\varphi_{3}GDP_{t}+\varphi_{4}HE_{t}\\ \;+\varphi_{5}Internet_{t}+\varepsilon_{t} \end{array}$$

Specification (1) is the health function that depends on financial development (FD), financial globalization (FG), gross domestic product (GDP), health expenditure (HE), and internet users (Internet). We have employed life expectancy as a proxy of health outcomes. Claessens and Feijen (3) reported that financial development positivity affects life expectancy *via* various channels such as income, education, gender equality, infrastructure, and clean energy effects, thus estimate of *φ*_1_ could be positive. Similarly, financial globalization is a source of capital inflows, which improves health outcomes. Consequently, an estimate of *φ*_2_ is expected to be positive. Economic development, health expenditure, and the internet are possible key determinants of life expectancy observed in the field of health economies, thus estimates *φ*_3_, *φ*_4_, and *φ*_5_ are expected to be positive. On the other hand, *φ*_0_ represents constant term and εt is the white noise error term. The basic model has given us only long-run estimates by ignoring short-run. To differentiate the long-run impacts of financial development and financial globalization on health from its short-run effects, again following Pesaran et al. (25) we express specification (1) in an error-correction formwork as in specification (2) below:


(2) $$\begin{array}{l} \text{Δ}Health_{t}=\;\omega_{0}+\;\sum_{k=1}^{n}\beta_{1k}\text{Δ}Health_{t-k}+\sum_{k=0}^{n}\beta_{2k}\text{Δ}FD_{t-k}\;\\ +\sum_{k=1}^{n}\beta_{3k}\text{Δ}FG_{t-k}+\sum_{k=0}^{n}\beta_{4k}\text{Δ}GDP_{t-k}+\sum_{k=0}^{n}\beta_{5k}\text{Δ}HE_{t-k}\;\\ +\sum_{k=0}^{n}\beta_{6k}\text{Δ}Internet_{t-k}+\;\varphi_{1}Health_{t-1}+\;\varphi_{2}FD_{t-1}+\varphi_{3}FG_{t-1}\;\\ +\;\varphi_{4}GDP_{t-1}+\varphi_{5}HE_{t-1}+\varphi_{6}Internet_{t-1}+\;\varepsilon_{t} \end{array}$$

Specification (2) is similar to Engle and Granger (26). It is labeled linear ARDL model. Therefore, Equation (2) outlines a one-step estimation process that yields the long-run and short-run effects of concern variables on health (27, 28). The estimates deliver short-run impacts of the coefficients devoted to “Δ” variables. The long-run impacts are gained by the estimates of *ω*_2_*ω*_6_ normalized on *ω*_1_. We can assess cointegration for the validity of estimates by using an F-test and ECM or *t*-test. The null hypothesis of the *F*-test in the equation is H0: *φ*_1_ = *φ*_2_ = *φ*_3_ = *φ*_4_ = *φ*_5_ = *φ*_6_= 0, which infers the non-existence of long-run connection. While the alternative hypothesis is H1: *φ*_1_≠0, *φ*_2_≠0, *φ*_3_=0, *φ*_4_≠0, *φ*_5_≠0, *φ*_6_≠0. ARDL model assuming all variables to be integrated of either I(0) or I(1), even blend of two. This approach accommodates I(0) and I(1) variables, and it is also robust to small samples. To confirm the model's goodness of fit, some diagnostic and stability tests are applied. For instance, testing for serial correlation (LM), functional form (RESET), heteroscedasticity (BP) is robustly linked with the selected model. We can use cumulative sum (CUSUM) and cumulative sum of squares (CUSUM-sq) for stability. The next section can estimate Equation (2) for three larger Asian economies.

### Data

The study examines the impact of financial development and financial globalization on the health outcomes of selected Asian economies from 1990 to 2019. These economies contain the sample of China, India, and Japan. Table 1 provides details regarding definitions, symbols of variables, and sources of data. The dependent variable, health outcome, is measured by life expectancy at birth. Previous literature explored the effect of GDP, health expenditure, and ICT on health (29, 30). Based on standard literature, we explore the importance of financial development and globalization on health by controlling GDP, health expenditure, and ICT variables. However, the financial development index and financial globalization index are major focused variables. The list of control variables consists of GDP per capita (at constant 2010 US$), current health expenditures (percent of GDP), and internet users (in percent of the population). Data for the financial development index is obtained from IMF. Data for the financial globalization index is taken from KOF Swiss Economic Institute. However, data for life expectancy, GDP, health expenditures, and the internet is extracted from the World Bank. In Table 1, the average life expectancy in China, Japan, and India was 72.94, 81.87, and 64.45 with mean FDI (FG) of 0.459 (3.700), 0.747 (3.950), and 0.389 (3.386), respectively.

**Table 1:** Data definitions, description, and sources.

**Variables**	**Definitions**	**China**	**Japan**	**India**	**Sources**
Health	Life expectancy at birth, total (years)	72.94	81.87	64.45	World bank
FDI	Financial development index	0.459	0.747	0.389	IMF
FG	Financial globalization index	3.700	3.950	3.386	KOF Swiss Economic Institute
GDP	GDP per capita (constant 2010 US$)	7.929	10.68	6.974	World bank
HE	Current health expenditure (% of GDP)	4.623	8.097	3.823	World bank
Internet	Individuals using the Internet (% of population)	20.69	52.22	6.577	World bank

## Results and Discussion

Before performing regression analysis, the study confirms the order of integration for life expectancy, financial development index, financial globalization index, GDP, health expenditures, and internet. For that purpose, the study employs two-unit root tests: Augmented Dickey-Fuller (ADF) and Phillips Perron (PP) tests. Table 2 presents the results of both unit root tests. The test statistics show that GDP variables are stationary at the level and that all other variables are first difference stationery in China. In the case of Japan, FDI is level stationery, and all other variables are first difference stationary. In the case of India, life expectancy and financial development index are level stationery and all other variables appear stationary at first difference. Next, the study employs the ARDL approach to explore the impact of financial development and financial globalization on CO_2_ emissions. Table 3 presents the short-run and long-run coefficient estimates of all three ARDL models for China, Japan, and India.

**Table 2:** Unit root test.

		**ADF**			**PP**		
		**I(0)**	**I(1)**	**Decision**	**I(0)**	**I(1)**	**Decision**
China	Le	1.468	−3.446^**^	I(1)	0.816	−2.877^*^	I(1)
	FDI	−0.701	−5.131^***^	I(1)	−0.691	−5.129^***^	I(1)
	FG	−1.774	−5.088^***^	I(1)	−1.891	−5.077^***^	I(1)
	GDP	−3.399^***^		I(0)	−2.826		I(0)
	HE	−0.966	−3.514^**^	I(1)	−1.272	−3.445^**^	I(1)
	INTERNET	3.563	−2.678^*^	I(1)	1.568	−2.766^*^	I(1)
Japan	Le	−0.803	−6.889^***^	I(1)	−0.944	−7.322^***^	I(1)
	FDI	−2.752^*^		I(0)	−2.812^*^		I(0)
	FG	0.631	−3.969^***^	I(1)	0.597	−3.912^***^	I(1)
	GDP	−0.476	−5.427^***^	I(1)	−0.291	−5.476^***^	I(1)
	HE	−0.234	−4.541^***^	I(1)	−0.255	−4.593^***^	I(1)
	INTERNET	−1.097	−3.663^**^	I(1)	−0.973	−3.651^**^	I(1)
India	Le	−6.957^***^		I(0)	−3.926^***^		I(0)
	FDI	−2.838^*^		I(0)	−2.870^*^		I(0)
	FG	−1.336	−3.572^**^	I(1)	−1.331	−3.605^**^	I(1)
	GDP	1.572	−4.241^***^	I(1)	1.930	−4.183^***^	I(1)
	HE	−1.316	−4.453^***^	I(1)	0.805	−4.438^***^	I(1)
	INTERNET	0.546	−2.875^*^	I(1)	0.654	−2.678^*^	I(1)

*** *p < 0.01*;

** *p < 0.05; and*

* *p < 0.1*.

**Table 3:** Short and long-run estimates of ARDL.

	**China**		**Japan**		**India**	
**Variable**	**Coefficient**	***t*** **-Stat**	**Coefficient**	***t*** **-Stat**	**Coefficient**	***t*** **-Stat**
**Short-run**
D(FDI)	0.010^**^	2.439	0.598	0.279	0.187^*^	1.883
D(FDI(-1))					0.159	1.438
D(FG)	0.015	1.262	0.626	0.723	0.039	0.430
D(FG(-1))			−1.645	2.145	−0.152	2.360
D(GDP)	0.135^*^	2.030	0.778	0.253	0.332	1.487
D(GDP(-1))	0.127	1.573	−2.777	1.968	−0.475^*^	1.690
D(HE)	0.003	0.413	0.004	0.038	0.105^***^	3.605
D(HE(-1))					0.039^**^	2.475
D(INTERNET)	0.000	0.037	0.011^*^	1.704	0.001	1.291
D(INTERNET(-1))					0.015^**^	2.214
**Long-run**
FDI	0.599^*^	1.787	0.733	0.283	1.235	1.180
FG	0.243	1.208	1.247^*^	1.796	1.121^**^	2.135
GDP	2.614^***^	12.93	1.842^***^	3.638	1.060	0.524
HE	0.042	0.412	0.252^***^	3.604	0.166^*^	1.765
INTERNET	0.019^***^	5.011	0.013^***^	2.083	0.076	1.002
C	5.662^***^	38.03	7.905^*^	1.911	−75.61	0.582
**Diagnostics**
*F*-test	12.32^***^		4.654^***^		8.652^***^	
ECM(-1)	−0.631^***^	16.85	−0.716^***^	4.806	−0.568^**^	1.994
LM	1.856		1.489		1.425	
BP	0.845		0.365		0.547	
RESET	0.223		1.452		1.023	
CUSUM	S		S		S	
CUSUM-sq	S		S		S	

*** *p < 0.01*;

** **p < 0.05; and

* *p < 0.1.

In the long-run, findings show that financial development has a positive and significant impact on life expectancy at a 10% level in China only, while the impact is statistically insignificant in the case of Japan and India. It shows that a 1% upsurge in financial development increases life expectancy by 0.599% in China. In our study, findings display that the impact of financial development on health outcomes is significant and positive. This means that easy access to finance through financial globalization enables people to choose a better and healthier lifestyle, foods, medical treatment, and accommodation. Meanwhile, financial development expands the life expectancy through the channels of gender equality, infrastructure, education, and per capita GDP. This finding is also supported by Levine (31), who noted that financial development alleviates poverty and promotes economic development by stimulating innovations, minimizing the cost of transactions, resource allocation in the production sector, and mobilization of savings, which improves life expectancy. This means that financial development positively impacts life expectancy and health through the channel of improvement in educational attainment (24). A possible reason is that educational attainment helps in generating more opportunities for educated people that ultimately improve the life expectancy and health outcomes of people. Our finding is also backed by Shahbaz et al. (32), who argued that financial development improves health outcomes through the channel of infrastructural impact. The findings suggested that financial development enables women to participate in income-generating activities and reduces gender inequality. Another reason is that self-employed women better take care of themselves and their children and invest more in healthcare services.

In contrast, financial globalization shows a positive and significant impact on life expectancy at 10% level in Japan and 5% level in India, while in the case of China, findings display insignificant results. It shows that in the long-run, a 1% upsurge in financial globalization increases life expectancy by 1.247% in Japan and 1.121% in India. These findings infer that the Chinese government can adopt financial development as a policy measure to enhance their life expectancy, while Japanese and Indian governments should adopt financial globalization as a policy tool to enhance their life expectancy level. Our study observed that the impact of financial globalization on health outcomes is significant and positive. This finding is also backed by Cornia (33), who argued that financial globalization positively affects life expectancy by easing the movement of pharmaceutical services and nutrition and providing technologies for proper sanitation, safe water for drinking, sufficient pharmaceuticals from emerging and advanced economies, and good quality medical treatment. This result is reliable with Dreher (13), who claimed that financial globalization enables economies to prosper and grow, indicating that financial globalization could be beneficial for economies' development and economic growth. This means that financial globalization influences health outcomes through education and income channels. Income channel states that financial globalization enhances the purchasing power of household. However, the education channel demonstrates that financial globalization expands life expectancy by enhancing literacy.

Gross domestic product displays a significant and positive impact on life expectancy in China and Japan at a 1% significance level. It shows that a 1% rise in GDP increases life expectancy by 2.614% in China and 1.842% in Japan in the long-run. Findings reveal that current health expenditures have a significant and positive impact on life expectancy at 1% level in Japan and 1% level in India. It infers that in the long-run, a 1% expansion in health expenditures increases life expectancy by 0.252% in Japan and 0.166% in India. Long-run findings display that the internet has a significant and positive effect on life expectancy at a 1% level in China and Japan, revealing that a 1% increase in the use of the internet increases life expectancy by 0.019% in China 0.013% in Japan.

The relationship between financial development and life expectancy is significant and positive at 5% in China and at 10% in India in the short-run. However, the impact of financial globalization on life expectancy is statistically insignificant in Asian economies in the short-run. The impact of GDP is positive and statistically significant on life expectancy at a 10% level in the short-run. Health expenditures have a significant and positive impact on life expectancy in India at a 1% level. At the same time, the internet has a significant and positive effect on life expectancy at a 10% level in Japan in the short run. The lower panel of Table 3 provides findings of some important diagnostic tests. It is found that long-run cointegration exists among variables in all three models, as shown by the expected findings of F-statistics and ECM term. No heteroskedasticity and autocorrelation are diagnosed in all three models as depicted by the results of BP and LM tests. It is found that error terms are normally distributed, and stability condition holds in all three models as revealed by the results of the Ramsey RESET test and CUSUM and CUSUM-sq tests.

## Conclusion and Policy Implications

The importance of life expectancy on economic growth is well established in the existing stock of literature. However, no study has documented the impact of financial globalization and financial development on health outcomes in Asian economies. Thus, our study aims to fill this vacuum using the ARDL approach to explore the long-run cointegration among financial globalization, financial development, and life expectancy. The study considers three Asian economies, namely China, Japan, and India, from 1990 to 2019. The study uses current health expenditures, GDP per capita, and the internet as control variables. The empirical findings indicate that financial development tends to improve life expectancy in China in the long-run, while it improves life expectancy in China and India in the short-run. In contrast, financial globalization improves life expectancy in Japan and India in the long-run. However, financial globalization produces no impact on life expectancy in the short-run. Moreover, health expenditures, GDP per capita, and the internet are positively associated with life expectancy in the long-run and short-run, revealing that these determinants positively contribute to enhancing the health outcome of individuals.

Our findings put forward some important policy implications for Asian economies. It is found that financial globalization and financial development improve health outcomes, suggesting that these two measures can be adopted as effective policy tools to enhance individuals' life expectancy. It is suggested that the governments and policymakers in Asian economies should not undervalue the role of financial globalization and financial development while designing health-related policies. Findings also suggest that financial sector improvement should be considered key to enhancing life quality, productivity, and economic growth for Asian economies. Findings reveal the positive impact of GDP per capita on life expectancy, suggesting that policymakers should adopt economic development to improve individuals' health outcomes. Moreover, digitalization shows positive effects on life expectancy, suggesting that smart technologies should be increased in the health sector. Additionally, current health expenditures in the government budget should be increased to improve individuals' life expectancy.

The study has identified some limitations, such as energy sector and environmental sector variables are not included in the analysis. Although, environmental and energy-related issues are highly affecting health outcomes. It is suggested that in future research, these variables should be included. Another future direction is that the impact of financial inclusion should be investigated for the health industry. A similar objective can also be tested for regional and group-wise economies in the future.

## Data Availability Statement

Publicly available datasets were analyzed in this study. This data can be found here: https://data.worldbank.org/.

## Author Contributions

GS: conceptualization, software, data curation, and writing—original draft preparation. DW: methodology, writing—reviewing, and editing. MA: visualization and investigation. All authors contributed to the article and approved the submitted version.

## Funding

This study was supported by Education Department of Jilin Province, Project Name: Research on Financial Risk Early Warning and Prevention in Jilin Province in the Post-Pandemic (Project Number: JJKH20211381SK).

## Conflict of Interest

The authors declare that the research was conducted in the absence of any commercial or financial relationships that could be construed as a potential conflict of interest.

## Publisher's Note

All claims expressed in this article are solely those of the authors and do not necessarily represent those of their affiliated organizations, or those of the publisher, the editors and the reviewers. Any product that may be evaluated in this article, or claim that may be made by its manufacturer, is not guaranteed or endorsed by the publisher.
